# Phytofabrication, characterization of silver nanoparticles using *Hippophae rhamnoides* berries extract and their biological activities

**DOI:** 10.3389/fmicb.2024.1399937

**Published:** 2024-07-24

**Authors:** Neha Rana, A. Najitha Banu, Bimlesh Kumar, Sandeep K. Singh, Noha E. Abdel-razik, Naif A. Jalal, Farkad Bantun, Emanuel Vamanu, Mahendra P. Singh

**Affiliations:** ^1^School of Bioengineering and Biosciences, Lovely Professional University, Phagwara, India; ^2^School of Pharmaceutical Sciences, Lovely Professional University, Phagwara, India; ^3^Indian Scientific Education and Technology Foundation, Lucknow, India; ^4^Department of Medical Laboratory Technology, Faculty of Applied Medical Sciences, Jazan University, Gizan, Saudi Arabia; ^5^Department of Microbiology and Parasitology, Faculty of Medicine, Umm Al-Qura University, Makkah, Saudi Arabia; ^6^Faculty of Biotechnology, University of Agricultural Sciences and Veterinary Medicine, Bucharest, Romania; ^7^Department of Zoology, DDU Gorakhpur University, Gorakhpur, India

**Keywords:** silver nanoparticles, *Hippophae rhamnoides*, green synthesis, optimization, antioxidant, lipid peroxidation, antimicrobial assay

## Abstract

**Introduction:**

Fabrication of plant-based metal nanoparticles has yielded promising results, establishing this approach as viable, sustainable, and non-toxic in the biomedical sector for targeted drug delivery, diagnostic imaging, biosensing, cancer therapy, and antimicrobial treatments.

**Methods:**

The present work demonstrates the suitability of *Hippophae rhamnoides* berries for the instant green synthesis of silver nanoparticles to check their antioxidant, lipid peroxidation, and antimicrobial potential. The preliminary characterization of *Hippophae rhamnoides*-mediated AgNPs was validated by monitoring the color shift in the solution from pale yellow to reddish brown, which was further confirmed by UV–vis spectroscopy and the plasmon peaks were observed at 450 nm. Field Emission Scanning Electron Microscopy (FESEM) and X-ray diffraction (XRD) were used to evaluate the surface topography and structure of AgNPs. Herein, the antioxidant potential of synthesized AgNPs was investigated using DPPH free radical assay and the antimicrobial efficacy of similar was checked against *E. coli* and *S. aureus* by following MIC (minimum inhibitory concentration) and MBC (Minimum bactericidal concentration) assay. Along with the inhibitory percentage of lipid peroxidation was analysed by following TBARS (Thiobarbituric acid reactive species) assay.

**Results & discussion:**

The results revealed that the AgNPs were spherical in shape with an average size distribution within the range of 23.5–28 nm and a crystalline structure. Negative zeta potential (−19.7 mV) revealed the physical stability of synthesized AgNPs as the repulsive force to prevent immediate aggregation. The bioactive functional moieties involved in reducing bulk AgNO_3_ into AgNPs were further validated by FTIR. TBARS was adapted to test lipid peroxidation, and *Hippophae rhamnoides*-mediated AgNPs showed a 79% inhibition in lipid peroxidation compared to *Hippophae rhamnoides* berries extract as 65%. Furthermore, the antibacterial tests showed 37 ± 0.01 mm and 35 ± 0.0132 mm, zones of inhibition against *E. coli* MTCC 1698 and *S. aureus* MTCC 3160 with MIC and MBC values of 1 mg/mL, respectively.

## 1 Introduction

The science of nanotechnology deals with the synthesis and manipulation of nanoparticles with at least one dimension that lies within the range of 100 nm or less. The fundamental structural component of a nanostructure is a nanoparticle, which is significantly smaller than the realm of everyday things but larger than individual atoms (Horikoshi and Serpone, 2013; Hawsawi et al., 2023) Metallic nanoparticles have revolutionized the field of biomedicine due to their small size (at least one dimension <100 nm) high conductivity, large surface-to-volume ratio, and surface plasmonic characteristics (Alex et al., 2020; Giri et al., 2022). These distinctive characteristics make them more profitable in composite fibers, electronic components, biosensing, cosmetic products, and antimicrobial applications along with making them an indispensable topic of research in electronics, chemistry, pharmaceutics, and medicine field. Among different nanometals, silver nanoparticles (AgNPs) have gained recognition as a result of their chemical stability, strong conductivity, and catalytic activity (Wang et al., 2016). AgNPs are well acknowledged for their broad-spectrum antimicrobial activity, anti-fungal and anti-cancer properties, and are currently being utilized in a variety of consumer products such as the food industry, household supplies, clothing, paints, and medical devices (Agnihotri et al., 2019; Kang et al., 2019; He et al., 2020; Ahmed et al., 2023).

AgNPs consist of 20 to 15,000 silver atoms and are usually less than 100 nm across (Yin et al., 2020). Due to the small dimensions and increased surface-to-volume ratio, silver nanoparticles have extraordinary antibacterial potential even at low concentrations at the same time non-toxic to humans (Rai et al., 2009). Through a comparative analysis of the antibacterial activity of AgNPs with different shapes, namely spherical, triangular, linear, and cubic, it is evident that the spherical AgNPs demonstrate a more pronounced and effective antibacterial impact (Hong et al., 2016; Xu et al., 2020). Multiple studies have demonstrated that there exists a positive correlation between the size of nanoparticles and their capacity to infiltrate bacterial cells (Bruna et al., 2021; More et al., 2023).

AgNPs have been synthesized involving physical synthesis (high temperature and pressure) or chemical synthesis (harmful chemicals) resulting in an impact on the environment and raising the overall cost of the process (Adebayo-Tayo et al., 2019). Contemporary to these methods, the last decade has emphasized on development of easy and facile biosynthetic green approaches for the production of AgNPs (Gul et al., 2021). The green approach has been favorably utilized to synthesize AgNPs employing the use of plant extracts and an array of microorganisms (bacteria, fungi, algae, yeasts, actinomycetes) eliminating the need for harmful compounds and allowing for expanded use in health care and pharmaceutical fields (Xu et al., 2020). However, the introduction of microorganisms remains challenging and has numerous downsides, such as the need to maintain an aseptic culture environment, proportionally lower production, and time-consuming as well as expensive processes (Chopra et al., 2022). In contrast, plants are abundant in the natural ecosystem and are easily available. They contain phytochemicals that can have the potential to serve as substitutes for chemical-reducing agents which are known to be highly toxic, expensive, and environmentally detrimental (Nguyen et al., 2022). Plants contain a wealth of biomolecules that can be utilized not only as medicines or nutraceuticals but also as multifaceted reagents in the green synthesis of metallic nanoparticles. Plants are the strong contenders for the one-pot synthesis of AgNPs where various biomolecules present in plants act as reducing agents. These reducing agents convert Ag^+^ ions to Ag^0^ during the nano synthesis. In addition, phytochemicals have demonstrated an important role as capping agents in the prevention of the aggregation of synthesized nanomaterials which enhance their stability and solubility (Xulu et al., 2022; Baroi et al., 2024). Hence, phytoconstituents can function as strong reducing and capping substrates, ensuring the stability of synthesized NPs.

**Figure 1:**
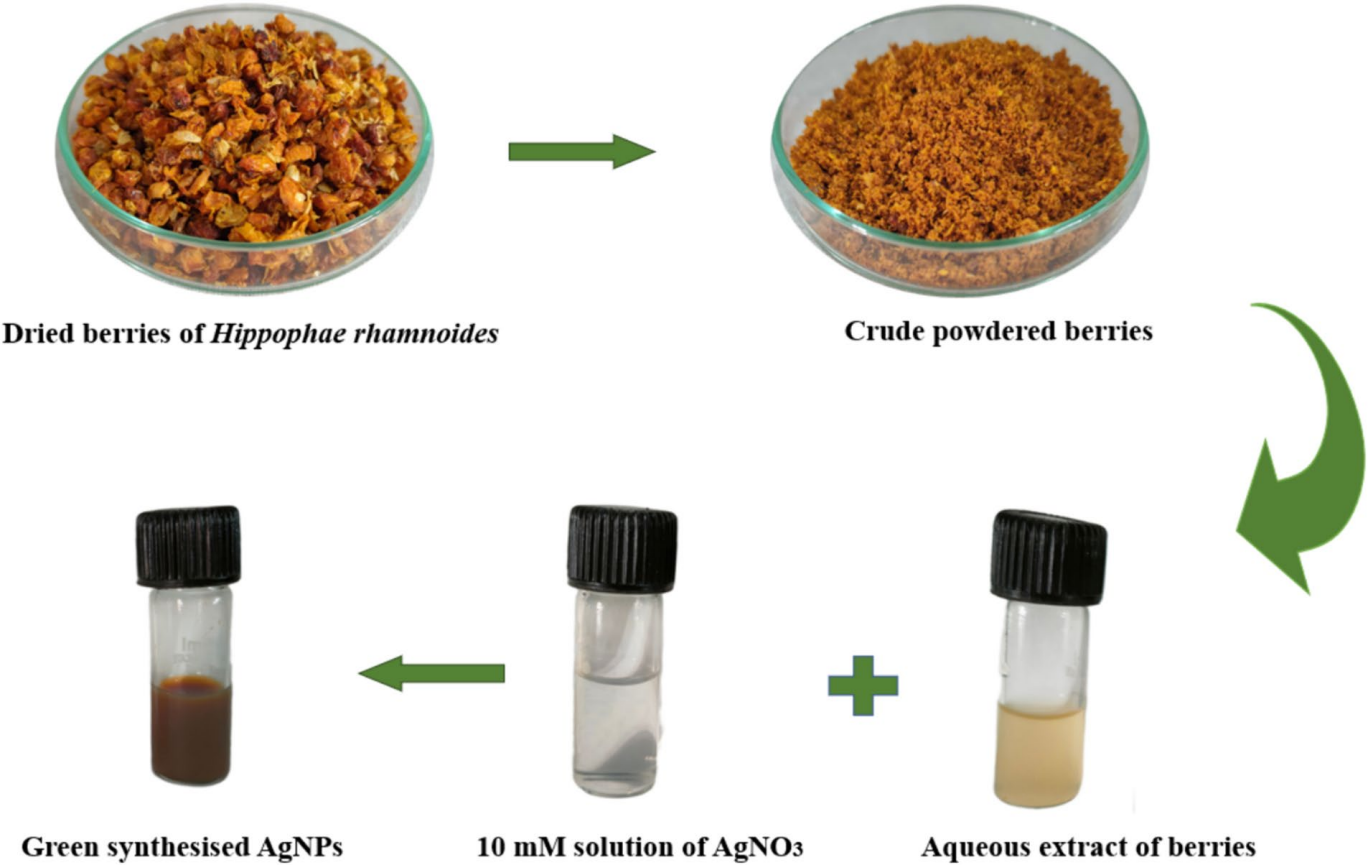
Pictorial representation of phytofabrication of AgNPs using *Hippophae rhamnoides*.

Three subsequent phases are involved in the synthesis of AgNPs from plant extracts. During the first phase, the metal ions (Ag^+^) get reduced into metal atoms (Ag^0^), followed by the nucleation of the reduced metal atoms. During the second phase, there occurs the accumulation of small adjacent AgNPs into larger particles, resulting in enhanced thermodynamic stability. The last phase, the termination phase, provides the final shape of the nanoparticles (Rana et al., 2023) (Figure 1). Plants are optimal for the synthesis of AgNPs due to diverse biomolecules such as flavonoids, terpenoids, and alkaloids, which act as effective reducing agents. According to Biju (2014), flavonoids undergo a tautomeric transformation from the enol to the keto form, which produces reactive hydrogen species that function as metal ion-reducing agents. Ketones and the carboxylic acid group of flavonoids participate in the reduction process of nanoparticles. Quercetin, a flavonoid, functions as a chelating agent is responsible for the bioreduction of metal ions.

Herein, we reported the use of *Hippophae rhamnoides* berries extract as a reducing and stabilizing agent for the fabrication of AgNPs (Figure 1). *Hippophae rhamnoides* (family Elaeagnaceae) frequently recognized as sea buckthorn (SBT) which is naturalized in temperate zones of Asia and Europe is a thorny nitrogen-fixing deciduous shrub with a plethora of pharmacological effects (Letchamo et al., 2018; Li et al., 2021; Giurescu et al., 2022; Geng et al., 2023). Berries of SBT are known to contain a high content of carbohydrates, proteins, vitamins, flavonoids (quercetin, isorhamnetin, kaempferol), and antioxidants (vitamins E & C) act as nutraceutical supplements with antibacterial, anti-allergic, anti-atherogenic and anticancer potential (Bal et al., 2011; Criste et al., 2020). *Hippophae rhamnoides* berries provide a wide range of health benefits, including antioxidant, anticancer, antiviral, antimicrobial, anti-hyperlipidemic, anti-obesity, anti-inflammatory, dermatological, neuroprotective, and hepatoprotective effects due to the presence of an array of phytochemicals (Luta et al., 2012; Jaśniewska and Diowksz, 2021). However, the pharmaceutical application of plant extracts is challenged by their lower bioavailability. In contrast, nanoparticles exhibit low molecular weights and are relatively soluble and chemically inert. These attributes offer nanoparticle-based drug delivery as a promising solution to this constraint in pharmaceutical development (Pramanik et al., 2021). Thus, *Hippophae rhamnoides* is suggested to be appropriate for the fabrication of AgNPs. These phytocompounds act as reducing, capping as well as stabilizing agents and have a propensity to absorb on nanoparticle surfaces making them biocompatible (Bharadwaj et al., 2021). These natural compounds reduce toxicity, improve cellular absorption, are stable to prevent aggregation and have therapeutic characteristics. The biogenic approach makes nanoparticles safer and more effective for biomedical purposes, making biological systems more accepting. AgNPs are also known to possess antibacterial, antiviral, and anti-inflammatory effects even at a low concentration. In this way, the synergistic effect of *Hippophae rhamnoides berries* and AgNPs has been observed, which is evident from the various applications used in paper such as antimicrobial potential, antioxidant activity, and inhibition of lipid peroxidation. The synthesis and stability of AgNPs are predominantly attributed to the abundance of polyphenolic functionalities in the extract. The present investigation intends to optimize the physical conditions for the fabrication of AgNPs utilizing sea buckthorn and access their antimicrobial potential.

## 2 Materials and methods

### 2.1 Reagents and chemicals

The reagents used to conduct the present research were of analytical grade including silver nitrate (AgNO_3_) solution, 2,2-diphenyl-1-picrylhydrazyl (DPPH) (99%), ascorbic acid, trichloro acetic acid, thiobarbituric acid, Sodium dodecyl sulfate (SDS), butanol, potassium chloride (KCl), acetic acid, streptomycin, Luria broth, and Mueller Hinton Agar were purchased from Loba Chemie.

### 2.2 Preparation of sea buckthorn berries extract

Dried berries of *Hippophae rhamnoides* were procured from Ladakh, India with latitude and longitude *coordinates*: 34.2268° N, 77.5619° E. The berries followed a process of rinsing with distilled water to remove impurities. Subsequently, 2 gm of crushed berries were boiled in 100 mL of distilled water for 40 min. The mixture was subjected to centrifugation and filtered using the Whatman filter paper no.1. The resulting filtrate was used as a reducing and stabilizing agent for the biogenic production of nanoparticles.

### 2.3 Biosynthesis of silver nanoparticles

The bio-fabrication of silver nanoparticles (AgNPs) is a one-pot synthesis in which 10 mL of plant extract was added to 90 mL of the freshly extracted solution of 10 mM silver nitrate (AgNO_3_) solution in a 1:9 ratio. For the effective synthesis of nanoparticles, different parameters were optimized, i.e., precursor concentration (1 mM, 2.5 mM, 5 mM, 7.5 mM, and 10 mM), the precursor: plant ratio (1,9, 1,5, 1,1, 5:1, 9:1) and pH (2–10). The change in color of the reaction mixture to the deep reddish brown signified the fabrication of AgNPs due to the reduction of Ag^+^ ions into AgNPs (Ashraf et al., 2016). Further, the reaction mixture was subjected to centrifugation at 10,000 rpm for a duration of 10 min. The resulting precipitates were rinsed with distilled water and finally with absolute ethanol. The subsequent precipitates were dried in the oven and utilized as AgNPs for further experiments.

### 2.4 Characterization of AgNPs

A UV–Vis spectrophotometer (Lasany Model No. LI-2800) was used to scan the absorption maxima of the reaction mixture between 200 and 800 nm to monitor the preliminary phases for the synthesis of reduced AgNPs in the reaction mixture. Distilled water was used as a blank. The Fourier-transform infrared spectroscopy; FTIR (Perkin Elmer Spectrum 2) of green synthesized AgNPs was performed in the range of 400–4,000 cm^−1^ with a resolution of 4 cm^−1^ using a spectrometer. AgNPs morphology was accessed by using Field emission scanning electron microscopy (FESEM) (FE-SEM: JEOL JSM-7610F). The crystallinity of the AgNPs produced was examined by X-ray diffraction (Bruker D8 Advance). The synthesized AgNPs were characterized by operating at 2θ from 30° to 80° at 0.041°/minute with a time constant of 2 s. Zeta potential was analyzed by Malvern Zetasizer Nano ZS90.

### 2.5 Antioxidant activity of AgNPs

The antioxidant efficacy of both SBT berry extract and AgNPs was evaluated by studying their free radical scavenging activity using the DPPH (2, 2-diphenyl-1-picrylhydrazyl) assay. In the process, 1 mL of test samples were individually mixed with 1 mL of DPPH. Five varied concentrations (4, 8, 12, 16, 20 mg/mL) of ascorbic acid, plant extract (SBT), and AgNPs were considered. This mixture was incubated for 30 min in dark conditions at 37°C. Subsequently, the concentration of radical was inquired by the reduction in absorbance percentage of the mixture at 517 nm wavelength. Ascorbic acid was employed as a reference or positive control. Methanol was used as a blank. Triplicates were tested to determine the % inhibition of DPPH free radicals using the absorbance of the control (A_c_) and test (A_t_) applying the following equation: (Brand-Williams et al., 1995).


$$\mathrm{Inhibition}\;\left(\%\right)=\left(A_{c}\right)-\left(A_{t}\right)/\left(A_{c}\right)*100.$$

### 2.6 Lipid peroxidation

The TBARS assay was used to quickly quantify the antioxidant properties of plant extract and green synthesized AgNPs. To quantify the lipid peroxide produced, egg yolk homogenates were used as a lipid-rich medium in a modified thiobarbituric acid-reactive species (TBARS) test (Dorman et al., 2000; Upadhyay et al., 2014). Two molecules of thiobarbituric acid (TBA) combine with malondialdehyde (MDA), a subsequent result of polyunsaturated fatty acid oxidation, resulting in a pinkish-red chromogen with an absorbance maximum at 532 nm. For the assay, 5% yolk material was prepared to a concentration of 10% (v/v) in KCl (1.15% w/v). After homogenizing for 30 s, the yolk was ultrasonicated for 5 min. Further, 0.1 mL of the test samples (plant extract, AgNPs) were added to SDS and subsequently to yolk homogenate. An aliquot of 1.5 mL of acetic acid [20% (v/v)] maintained at pH 3.5 was introduced to each sample to initiate lipid peroxidation. To the mixture, 1.5 mL [0.8% (w/v aqueous)] thiobarbituric acid (TBA) was added. Further, the addition of distilled water makes a total volume of 4 mL. Following 1 min of vortexing, the resulting mixture was subjected to 1 h of heating at 95°C. Following the cooling process, 5.0 mL of butanol was poured into each tube, and after 10 min of centrifugation at 3000 rpm, a maximal absorbance of 532 nm was noted. The control was provided only with TBA and distilled water.


$$\%\mathrm{inhibition}=\left(1-T/C\right)\times 100.$$

Where, *T* represents the absorbance of the test samples, while *C* represents the absorbance of the entirely oxidized control.

### 2.7 Minimum inhibitory concentration

The Minimum Inhibitory Concentration (MIC) is a quantitative technique utilized to ascertain the minimum concentration of an antimicrobial agent required to inhibit the growth of microorganisms. To determine the Minimum inhibitory concentration (MIC) of synthesized AgNPs and antibiotics, different concentrations of AgNPs and antibiotics were tested against *E. coli* and *S. aureus* as the methodology suggested by Parvekar et al., 2020. In this experiment, a range of 0.1 mg/mL–2 mg/mL (0.1, 0.5, 1.0, 1.5 and 2.0 mg/mL) AgNPs and streptomycin were added to the Mueller Hinton broth containing 100 μL of bacterial culture (24 h grown culture). The test samples were incubated at 37°C for 24 h in the incubator. The next day, the visible turbidity was checked to evaluate the growth of bacterial culture followed by taking optical density (OD) at 600 nm using a UV–vis spectrophotometer. The positive and negative controls were also tested. The complete study was conducted in triplicates. The percent growth inhibition was calculated by using the formula:


$$\mathrm{Percent}\;\mathrm{growth}\;\mathrm{inhibition}=\frac{OD\;\mathrm{of}\;\mathrm{control}-OD\;\mathrm{of}\;\mathrm{test}\;\mathrm{sample}}{OD\;\mathrm{of}\;\mathrm{control}}*100.$$

### 2.8 Minimum bactericidal concentration

The Minimum Bactericidal Concentration (MBC) is the lowest concentration of an antimicrobial agent that eliminates bacteria in the inoculum. To perform the MBC, 50 μL of bacterial culture was spotted on MHA plates by taking the inoculum from the respective test tubes. Different test concentrations and positive control (antibiotic) for *E. coli* and *S. aureus* were performed on the same plate. The plate was incubated at 37°C for 24 h in the incubator. The bacterial growth was evaluated by visualizing the colony obtained on the plate.

### 2.9 Antibacterial activity

The disc diffusion method was performed to test the antibacterial activity using Mueller Hinton agar (MHA) media as suggested by Balouiri et al. (2016) with a slight modification. An autoclaved sterilized media was prepared and poured into sterilized petri plates and set aside to solidify in a laminar flow. Gram-positive bacterial strain *Staphylococcus aureus* and gram-negative bacterial strain *Escherichia coli* were tested for use *in vitro* antimicrobial potential of biosynthesized AgNPs. The bacterial strains were cultured in Luria broth for 24 h at 37°C. The test species were spread over the Mueller Hinton agar media. Sterile blank antimicrobial susceptibility disks were used in the test. Further, the disks were loaded with 100 μL of (1 mg/mL) AgNPs, plant extract, AgNO_3_, distilled water, and streptomycin. Zones of inhibition (mm) of *Hippophae rhamnoides* mediated silver nanoparticles were resolute by utilizing the disk diffusion method.

## 3 Results

### 3.1 UV–vis spectroscopy analysis

**Figure 2:**
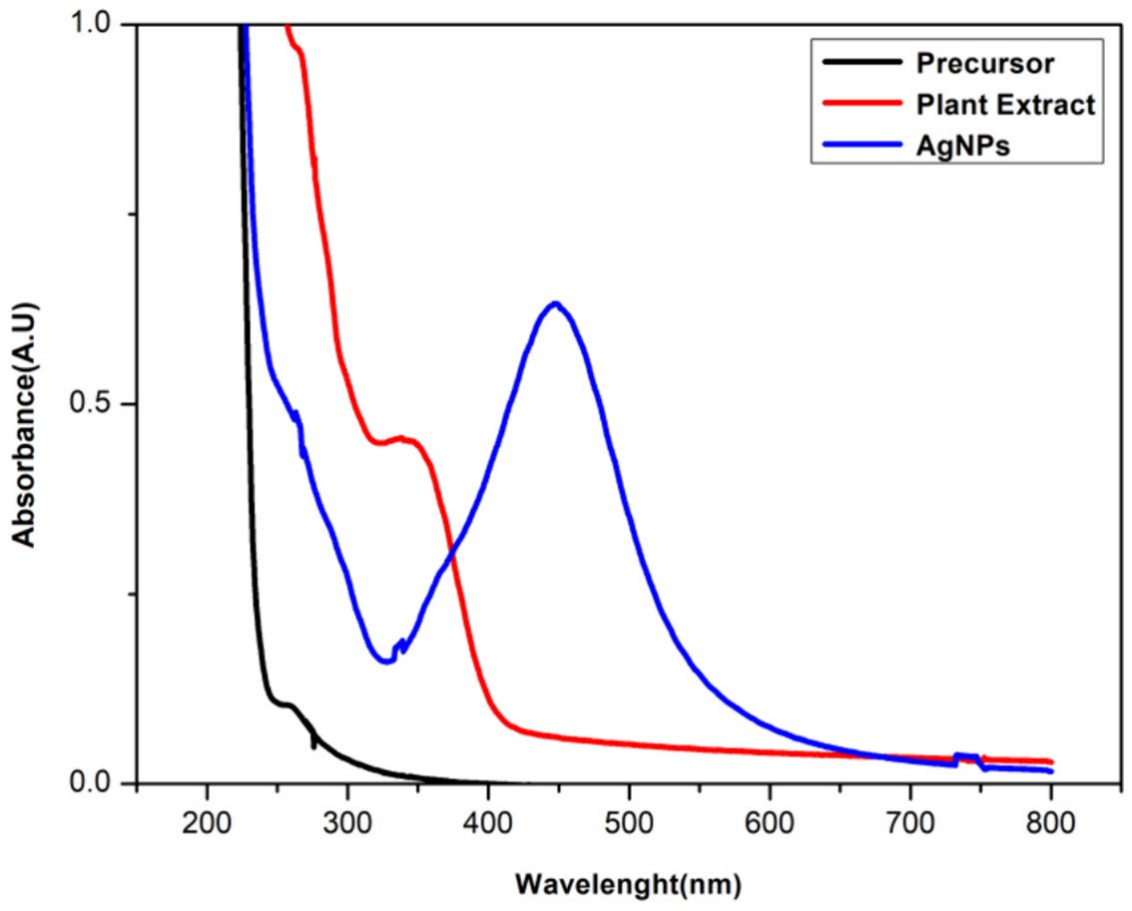
UV–vis spectrum of green synthesized AgNPs, plant extract, and AgNO_3_ (precursor).

To carry out the preliminary examination of silver nanoparticle synthesis, UV–vis spectrophotometric analysis was used. From the spectral analysis, a peak was observed at 450 nm which was found to be stable for a few days with no increase in absorption (Figure 2). The various factors that could affect the fabrication, shape, and size of AgNPs were optimized.

#### 3.1.1 Precursor concentration

**Figure 3:**
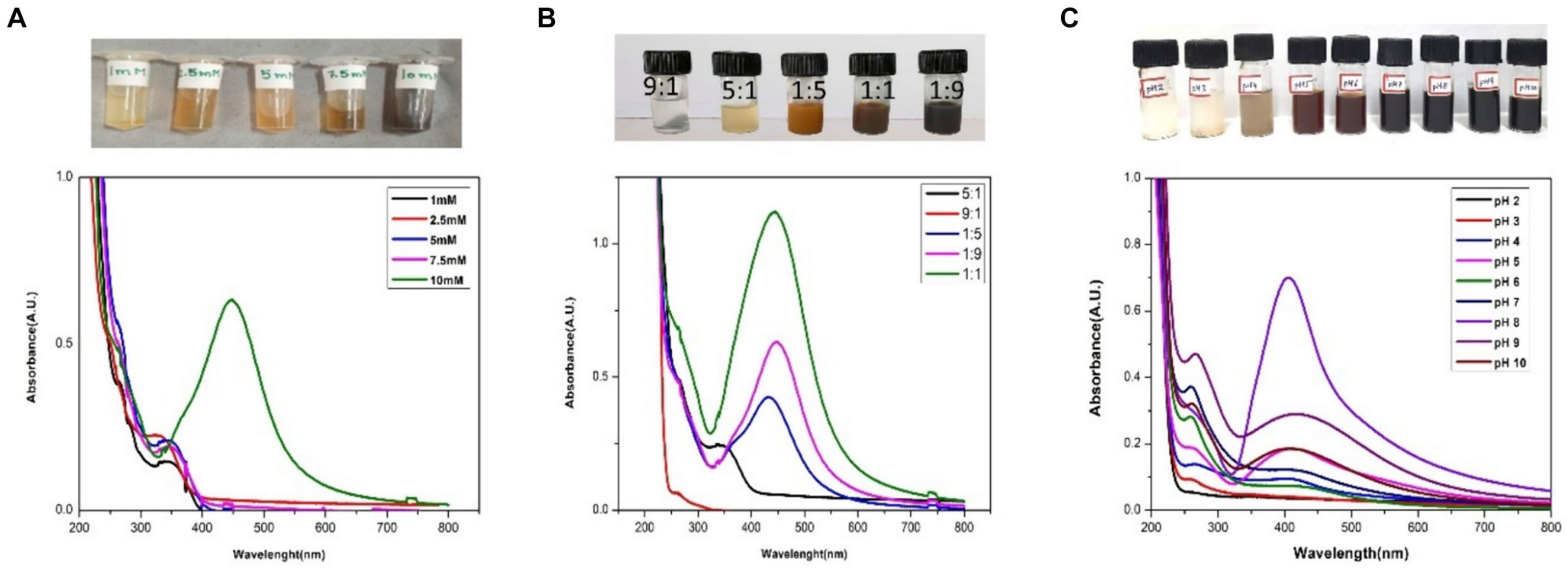
UV–vis spectra of the AgNPs optimization: **(A)** different concentrations of AgNO_3_ (1 mM, 2.5 mM, 5 mM, 7.5 mM and 10 mM); **(B)** different Plant Extract: AgNO_3_ ratios (9:1, 5:1, 1:1, 1:5, 1:9), and **(C)** different pH (2,3,4,5,6,7,8,9,10).

To check the optimal concentration of precursor for effective synthesis, five different concentrations of 1 mM, 2.5 mM, 5 mM, 7.5 mM, and 10 mM were added separately to 2% of the plant extract. SPR band was highest at 10 mM and showed the maximum absorbance at 10 mM (Figure 3A).

#### 3.1.2 Precursor and plant ratio

The ratio (precursor: plant extract) is another prime factor affecting the fabrication of nanoparticles. Different ratios (1:9, 1:5, 1:1, 5:1, 9:1) were taken and the synthesis of AgNPs was confirmed by color change and UV–vis analysis. According to the color change, 1:9, 1:5, 1:1 had shown the instant synthesis of AgNPs. By considering the UV–vis spectroscopy graph, 1:9 was considered the optimal ratio showing better SPR (Figure 3B).

#### 3.1.3 Effect of pH

The pH plays a vital role in nanoparticle synthesis, which can be considered the main parameter. After finalizing the molarity and ratio of precursor and plant extract, the reaction mixture was maintained for a range of pH from 2–10 and was optimized with 0.1 N hydrogen chloride (HCl) and 0.1 N sodium hydroxide (NaOH). It has been found that pH 8 was the best fit for the instant synthesis of AgNPs at room temperature with immediate color change (Figure 3C).

### 3.2 Fourier transform infrared spectroscopy

**Figure 4:**
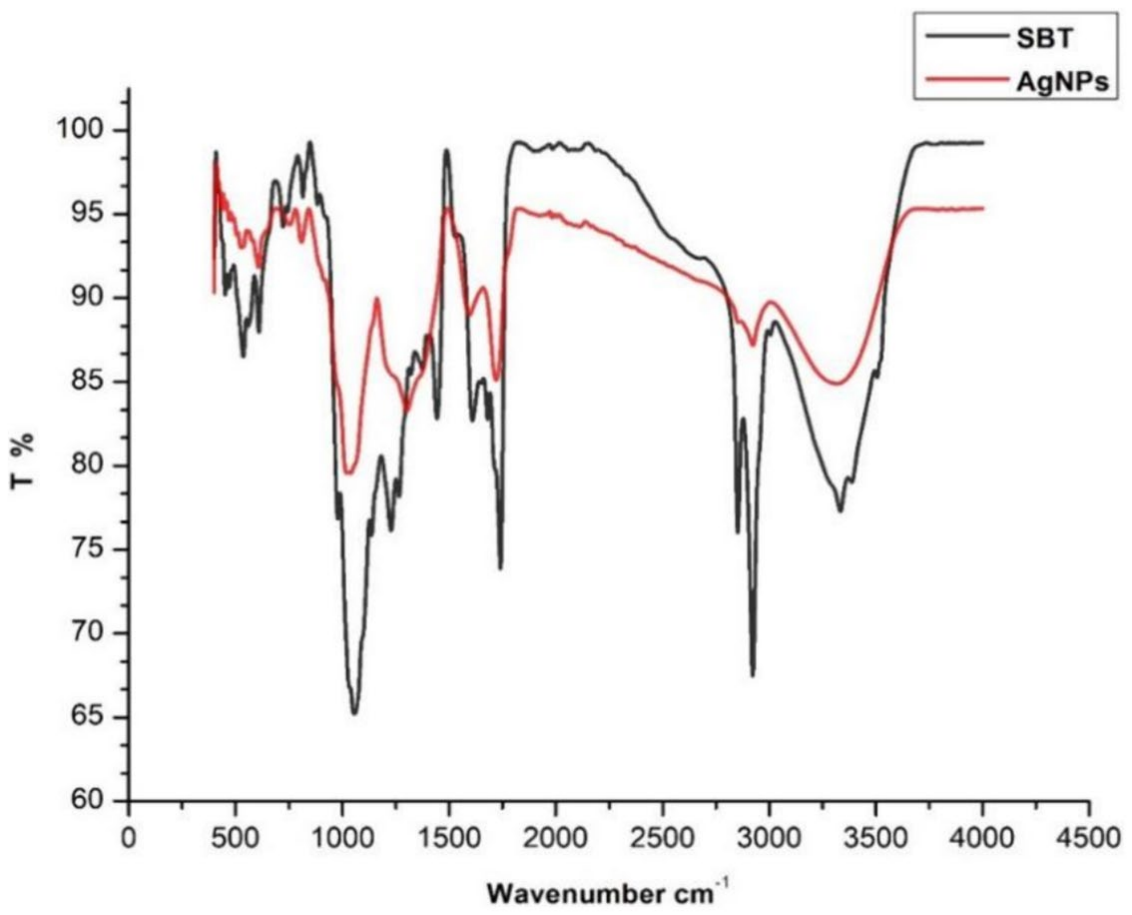
FTIR spectra of dried powder of *Hippophae rhamnoides* berries and silver nanoparticles synthesized by *Hippophae rhamnoides*.

The availability of potential functional groups in berries participating in the reduction of Ag ions as well as the capping and stabilization of synthesized AgNPs was examined using FTIR spectroscopy. The spectra of SBT berries extract and SBT-mediated AgNPs were documented in the 3,600–400 cm^−1^ range (Figure 4). The FTIR spectrum of sea buckthorn berries extracts represent the peaks at 3339, 2926, 1724, 1,603, 1,310, 1,043, and 637 cm^−1^. After the synthesis process of AgNPs, the peaks have shifted to higher wavenumbers representing the presence of capping agents with the nanoparticles. These changes in the stretching bonds of SBT-AgNPs could be related to the reduction, stabilization, and capping of nanoparticles. The shifting of peak indicated that the functional groups were active in the binding mechanism on AgNPs. The presence of strong to medium peaks around 3,339 cm^−1^ in extract and AgNPs ascribed to the O-H stretching vibrations of alcohols, phenols, carbohydrates, or even water in the case of aqueous extracts (Kalaiyarasan, 2022). Another peak was observed around 2,900 due to C-H stretching and the particular wavenumbers indicate the existence of predominantly aliphatic C-H bonds in fatty acids and pectin. The sharp peak around 1700 cm^−1^ hinted at the appearance of the (C=O) carbonyl group of flavonoids and lipids in plant extract and newly synthesized AgNPs (Oliveira et al., 2016). Peaks from 1750–1,600 cm^−1^, the two intense peaks viewed at 1660 cm^−1^ and 1,553 cm^−1^ indicated the presence of amides (Skotti et al., 2014). The bands around 1,613 cm^−1^ signify the presence of benzene which justified the presence of a phenolic group in SBT berries (Heredia-Guerrero et al., 2014). Based on the data presented above, polyphenolic compounds and proteins participated in the reduction of Ag^+^ to Ag^0^ and the stabilization of the nanoparticles (Figure 4).

### 3.3 X-ray crystallography

**Figure 5:**
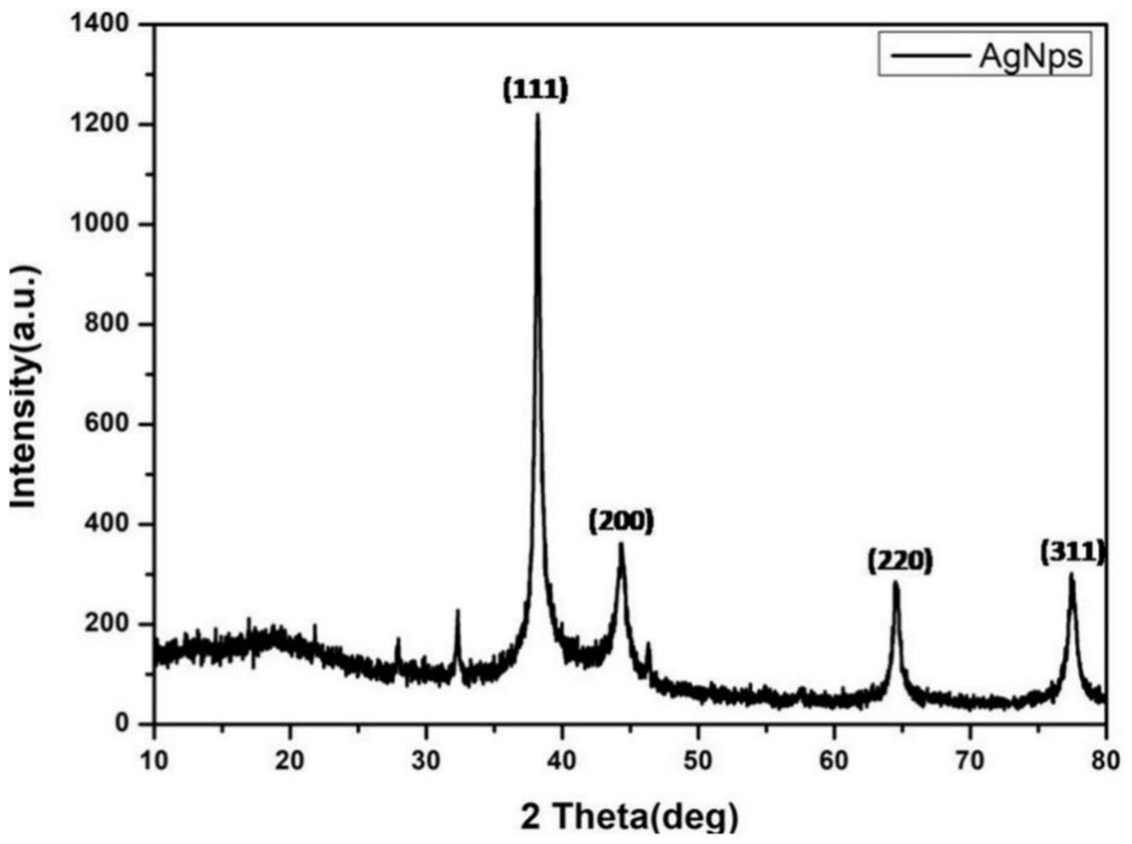
X-ray crystallography of AgNPs indicating the FCC structure.

The XRD pattern of AgNPs exhibited the appearance of four characteristic diffraction peaks at 38.1, 44.2, 64.4, and 77.6 with corresponding crystallographic planes indexed at (111), (200), (220), and (311) suggesting the onset of the face-centered cubic crystalline (FCC) AgNPs (Preda et al., 2020). The peak corresponding to the (111) plane is the sharpest, indicating that nanoparticles are most likely to show growth in this direction (Figure 5). The crystallinity of the synthesized AgNPs was evaluated using the Debye–Scherrer equation (Warren, 1941);


(1) $$D=\;\frac{K\lambda \;}{\beta \cos\theta }$$

Here, *D* represents the crystalline size of NPs, *K* is Scherrer constant (0.98), *λ* and *β* denotes the wavelength (1.54) and fullwidth at half maximum in radians (FWHM), θ is Bragg angle. The mean crystalline size of synthesized AgNPs was calculated to be 13.7 nm by following Eq. 1.

### 3.4 Field-emission scanning electron microscopy

**Figure 6:**
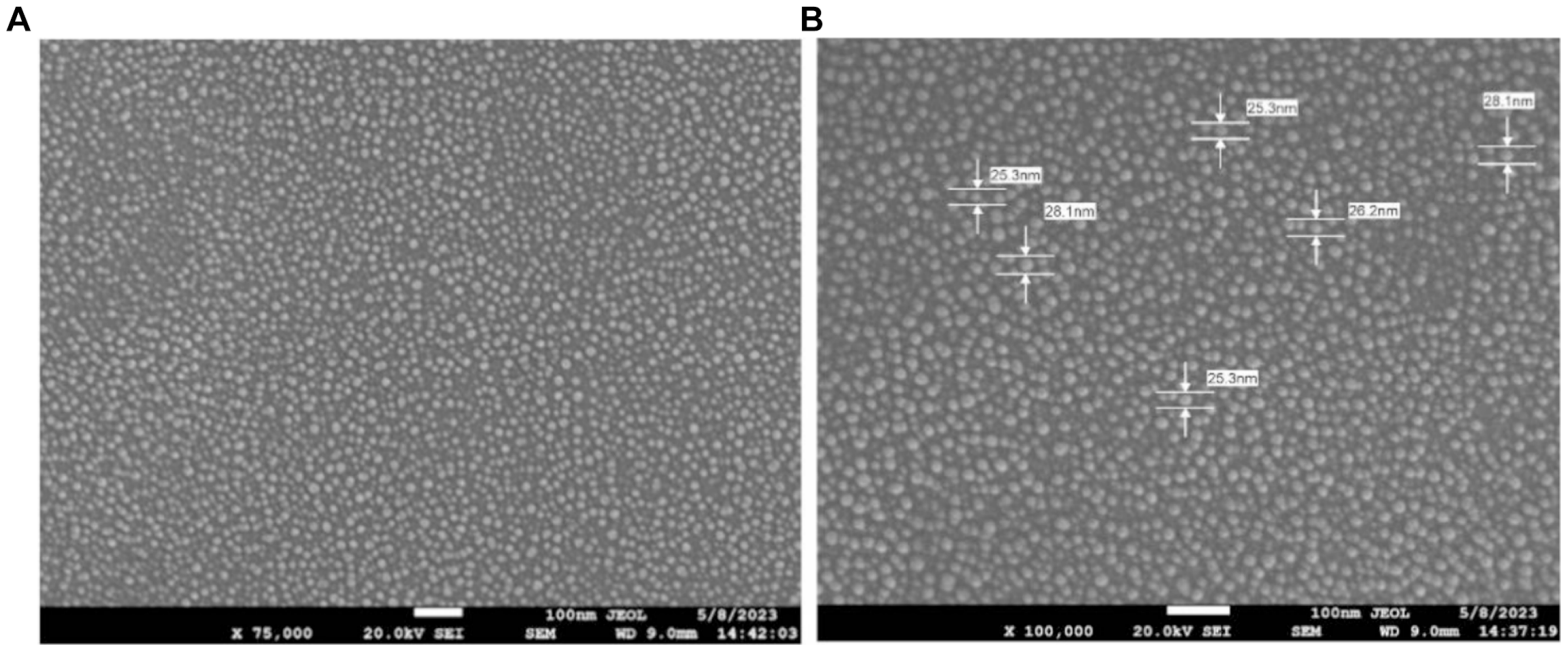
FE-SEM images of AgNPs depicting the morphology at two different resolutions. **(A)** Scanning Electron micrographs of Sea buckthorn mediated AgNPs at 75,000× magnification. **(B)** FE-SEM of AgNPs at 1,00,000× magnification.

The structure and morphology of synthesized nanoparticles were explored by Field Emission Scanning Electron Microscope (FE-SEM). The FE-SEM images shown in (Figure 6) evidenced the spherical shape of AgNPs with an average size of 25–28 nm.

### 3.5 Zeta potential

**Figure 7:**
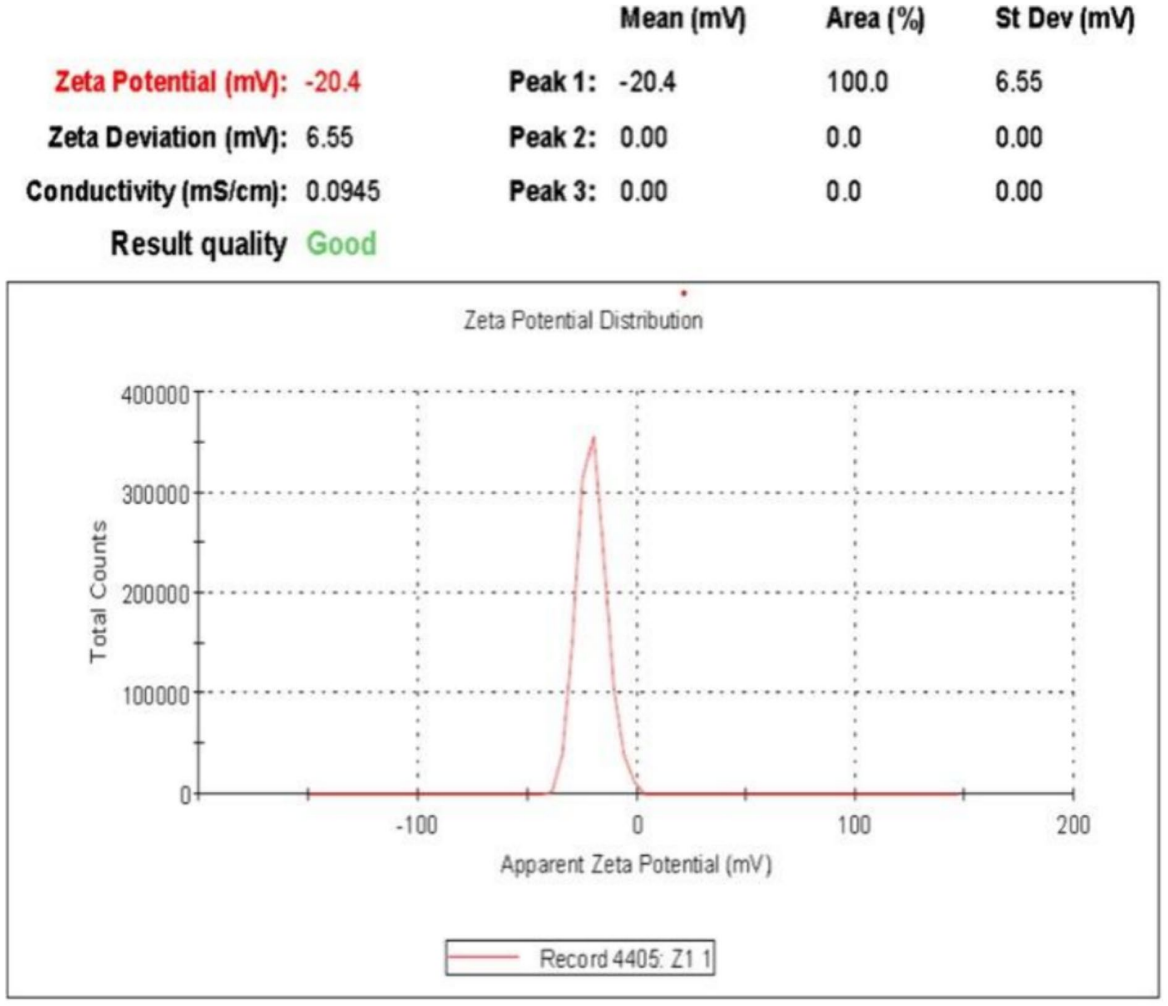
Zeta potential of biosynthesized AgNPs in aqueous medium using Zetasizer software.

The zeta potential of the silver nanoparticle solution derived from the SBT extract was measured to be −19.7 mV (Figure 7). This value indicates the presence of repulsion among the synthesized nanoparticles, as evidenced by the existence of a single peak.

### 3.6 DPPH radical scavenging assay

**Figure 8:**
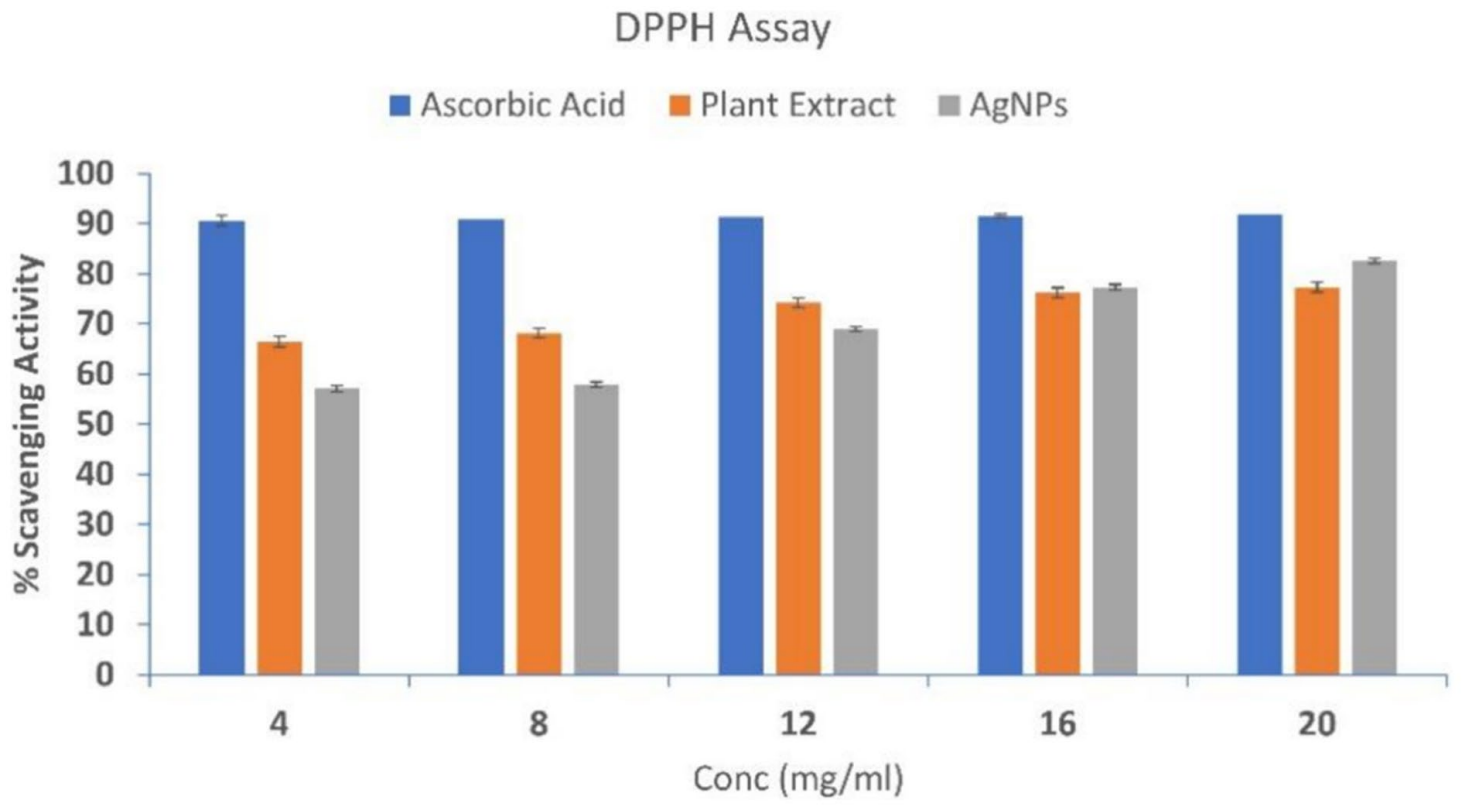
DPPH scavenging activity of Ascorbic Acid, Plant Extract, and AgNPs. At varied concentrations, the antioxidant potential was measured (4, 8, 12, 16, and 20 mg/mL). The DPPH scavenging activity was dose-dependent implying with the increase in concentration scavenging activity increases. The data are shown as mean ± SD for *n* = 3.

The antioxidant potential of SBT-mediated AgNPs was evaluated and compared to that of SBT extract and ascorbic acid (control) by their radical scavenging activity. The histogram suggests that the plant extract and AgNPs both exhibited free radical scavenging activity that was concentration-dependent (Figure 8). Out of five subsequent concentrations (4, 8, 12, 16, 20 mg/mL), 20 mg/mL showed the maximum % scavenging activity of 77% for SBT and 82% for AgNPs with DDPH.

### 3.7 Inhibition of lipid peroxidation (TBARS assay)

The inhibition of lipid peroxidation (Thio-Barbituric Acid-Reactive Species) of SBT and SBT fabricated AgNPs was determined by TBARS assay. The control was completely peroxidized whereas, plant extract and SBT-mediated AgNPs provide a degree of improvement in peroxidation indicated as 65 and 79% protection, respectively (Table 1).

**Table 1:** The percentage of lipid peroxidation inhibition of SBT berries and AgNPs using Egg yolk homogenate as media.

S. No.	Sample	% Inhibition (1 − T/C)*100
1	*Negative control	0
2	Plant extract	67.42 ± 1.20
3	AgNPs	76.78 ± 2.84

Values are expressed as the mean of triplicate measurements ± standard deviation (SD). *: No inhibitory agent was added to inhibit lipid peroxidation in negative control due to which it showed 100% peroxidation.

### 3.8 Minimum inhibitory concentration

**Figure 9:**
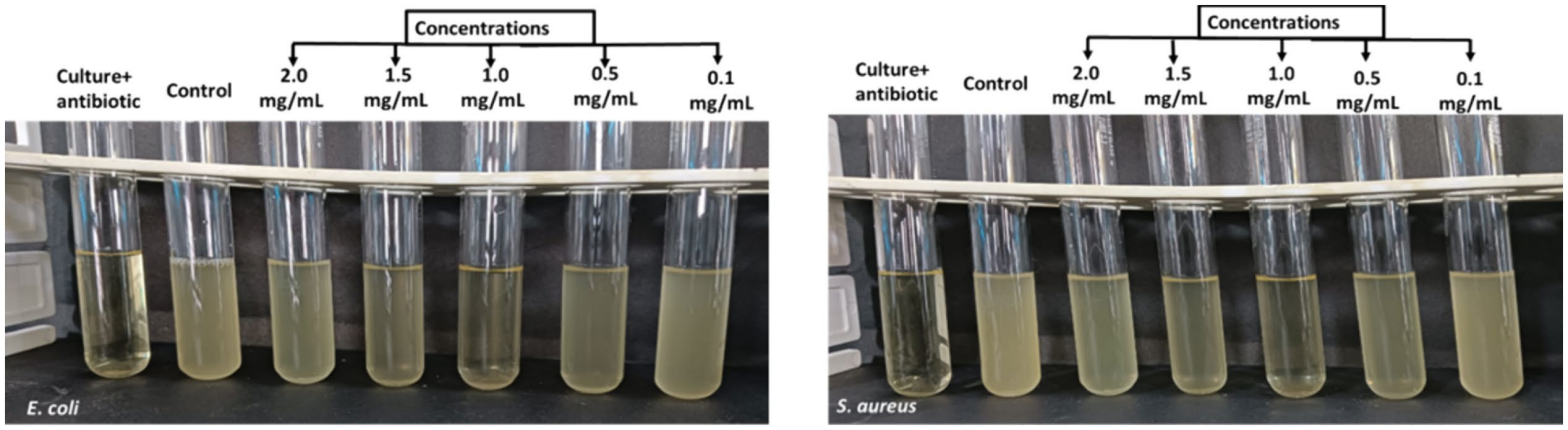
Determination of MIC of AgNPs (0.1 mg/mL, 0.5 mg/mL, 1.0 mg/mL, 1.5 mg/mL, 2.0 mg/mL) against *E. coli* and *S. aureus* depicting MIC at 1.0 mg/mL (lowest turbidity).

**Figure 10:**
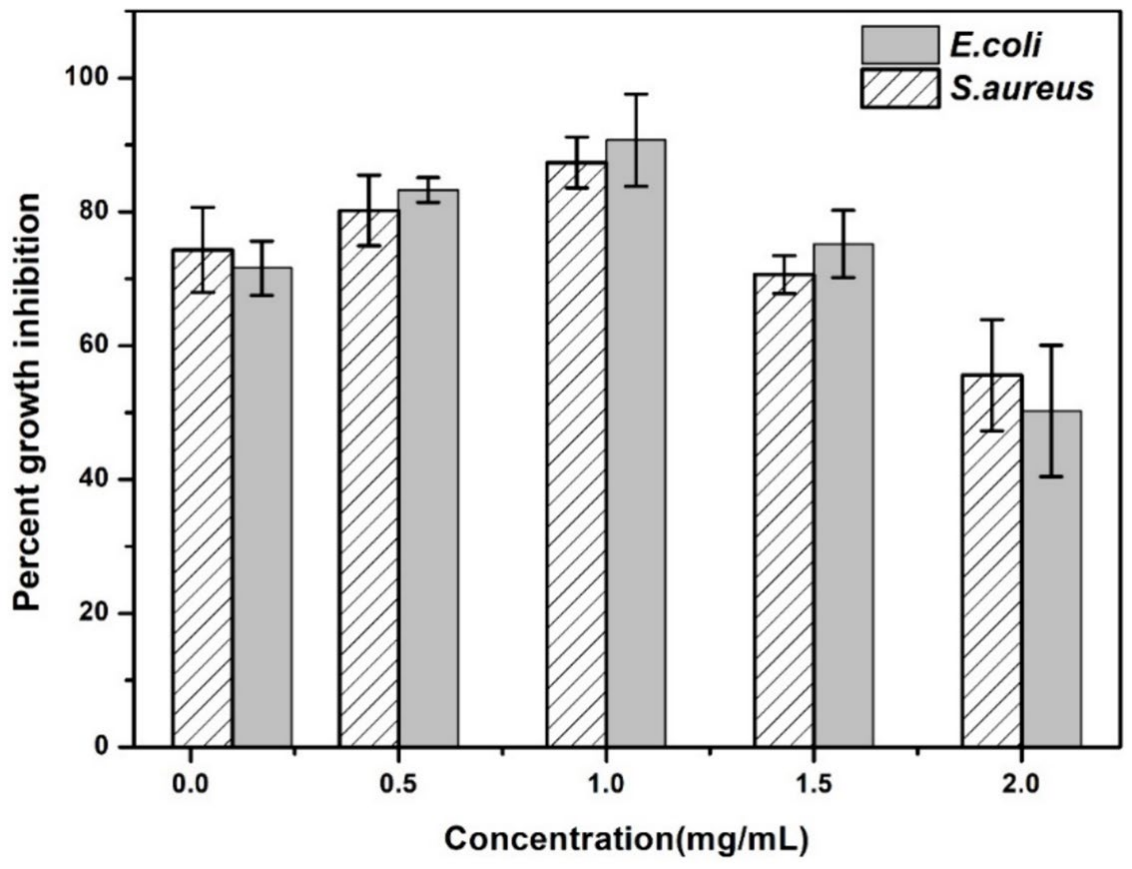
Percent growth inhibition of AgNPs at different concentrations (0.1 mg/mL, 0.5 mg/mL, 1.0 mg/mL, 1.5 mg/mL, 2.0 mg/mL) against the test strains *E. coli* and *S. aureus*. The data are shown as mean ± SD for *n* = 3.

The results obtained by visual observation showed that very low and higher concentrations of AgNPs could not inhibit bacterial growth effectively. From Figure 9, it is evident that enhancing the concentration of AgNPs up to a range reduced the bacterial turbidity exhibiting the inhibition of bacterial growth. Additionally, quantitative analysis has shown increasing the concentration of AgNPs from 0.1 mg/mL to 1 mg/mL inhibited the growth percent from 74.32 ± 6.35 and 71.58 ± 4.02% to 90.74 ± 6.88 and 87.37 ± 3.78% for *E. coli* and *S. aureus*, respectively (Figure 10). At the concentration of 1 mg/mL the maximum percentage growth inhibition for *E. coli* and *S. aureus* was calculated to be 90.74 ± 6.88 and 87.37 ± 3.78. On the contrary, increasing the range to more than 1 mg/mL, reduced the effectivity of AgNPs was observed, which is evident from Table 2. At the higher conentrations, the growth inhibition percentage was reduced approximately to 50% for both test samples. Hence, the final concentration chosen was 1 mg/mL based on visual observation and quantitative analysis.

**Table 2:** Percent growth inhibition of AgNPs at different concentrations against *E. coli* and *S. aureus*.

Concentration (mg/mL)	Percent growth inhibition (%)	
	*E. coli*	*S. aureus*
2 mg/mL	50.24 ± 9.79	55.59 ± 8.30
1.5 mg/mL	75.20 ± 5.02	70.62 ± 2.84
1 mg/mL	90.74 ± 6.88	87.37 ± 3.78
0.5 mg/mL	83.29 ± 1.84	80.21 ± 5.25
0.1 mg/mL	71.58 ± 4.02	74.32 ± 6.35

### 3.9 Minimum bactericidal concentration

**Figure 11:**
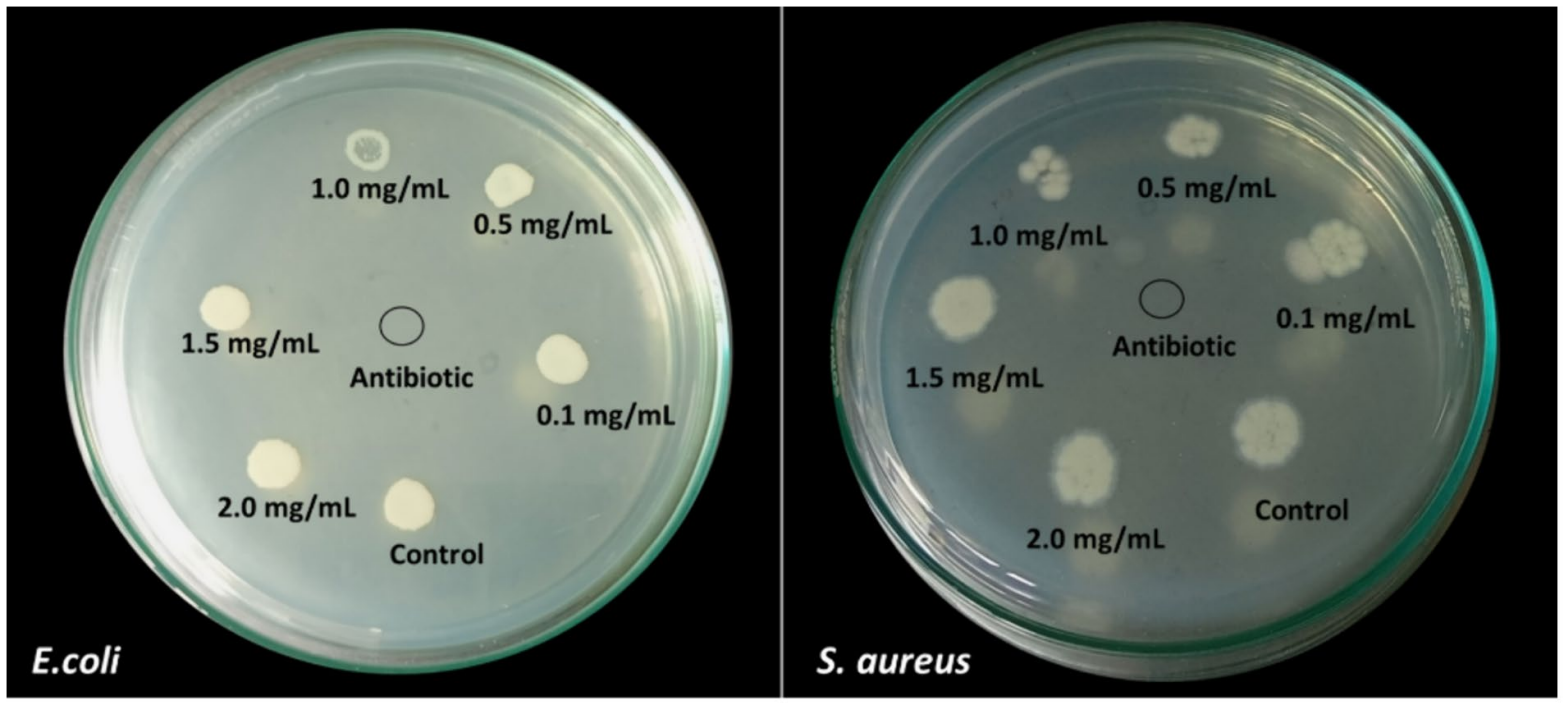
MBC results obtained from the different concentrations of AgNPs against *E. coli* and *S. aureus*.

In the present study, the MBC (minimum bactericidal concentration) was found to be the same as the MIC value. It is evident that 1 mg/mL is the optimal concentration to kill the bacterial growth by 90%. Along with, the reduction in the growth of microbial culture is visible from the MHA plates of *E. coli* and *S. aureus* (Figure 11). Even more, MIC and MBC have shown more sensitivity towards *E.coli* than *S. aureus*.

### 3.10 Antimicrobial assay

**Figure 12:**
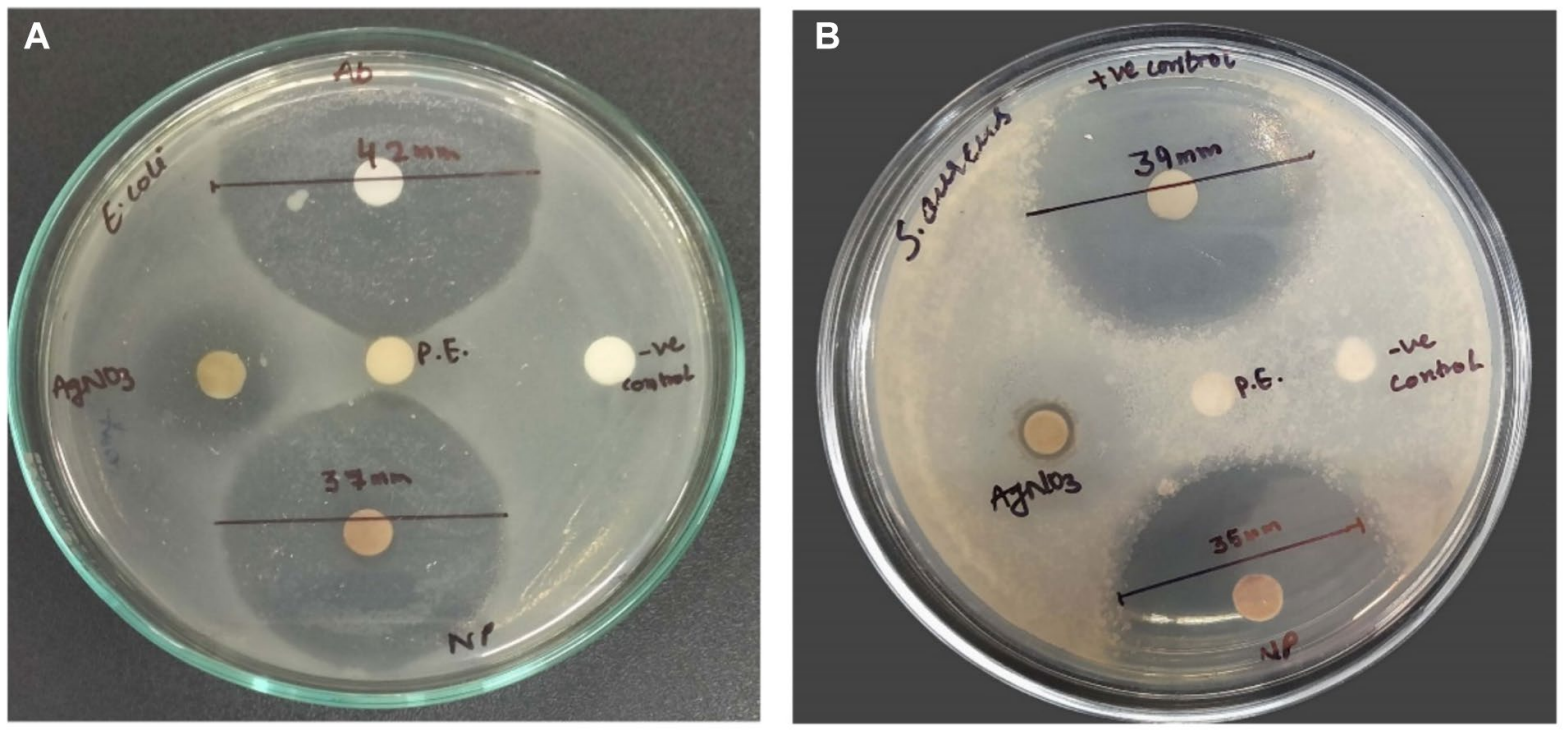
Pictures A and B show the antimicrobial activity of AgNPs, positive control (antibiotic), negative control (distilled water), plant extract (P.E.), and AgNO_3_ against *E. coli* and *S. aureus*, respectively. Data are shown as mean ± SD for *n* = 3. The mean difference is significant at the 0.05 level.

The *in vitro* antimicrobial activity of SBT-mediated AgNPs against the employed bacteria, was evaluated by the presence of inhibition zones and zone diameter. In our study, the antimicrobial activities of synthesized silver nanoparticles, AgNO_3_, plant extract, and distilled water were evidenced in comparison to standard antibiotic streptomycin. The zone of inhibition in the case of *E. coli* (MTCC 1698) and *S. aureus* (MTCC 3160) was reported to be 37 ± 0.0132 mm and 35 ± 0.0162 by AgNPs as compared to antibiotic streptomycin which showed a zone of inhibition 42 ± 0.032 mm and 39 ± 0.027, respectively, (Figure 12).

## 4 Discussion

The fabrication of NPs exploiting non-biological (physical and chemical) routes utilizes high energy, hazardous, and expensive chemicals. The following chemicals tend to be adsorbed on the surface of NPs making them non-biocompatible and toxic to the living system. By their toxicity, NPs synthesized using physiochemical methods have limited clinical applications in the pharmaceutical industry (Iqbal et al., 2021). In contrast to this plant-based nanoparticle (NP) synthesis is a benign, eco-friendly, and cost-effective revolutionary technique with numerous applications, in medicine, agriculture, and the food industry. Various bioactive compounds present in plant extracts used in green synthesis may enhance the therapeutic potential of nanoparticles. These compounds can significantly improve the overall process of drug distribution, circulation time, and distribution within the body and improve the efficacy of the treatment. Additionally, green synthesized nanoparticles have natural extract capping agents to stabilize and prevent agglomeration (Dowlath et al., 2021; Rana et al., 2023). In the current study, the synthesis of AgNPs has been executed using berries of sea buckthorn possesses a repertoire of biomolecules such as vitamins A, C, and E considerably high amounts of carotenoids, polyphenols, phytosterols, phenolic acids, and polyunsaturated fatty acids (Vilas-Franquesa et al., 2020; Gâtlan and Gutt, 2021). These phytochemicals have a promising role in the reduction, capping, and stabilization of AgNPs. This lends support to the previous findings in the literature where plant-based synthesis was preferred (Tang and Zheng, 2018; Cheng et al., 2020; Sharifi-Rad et al., 2021; Chandrasekharan et al., 2022). In the present synthesis, the addition of SBT extract to the AgNO_3_ solution resulted in a noticeable color change, shifting from a light-yellow hue to a deep reddish-brown. This alteration in color signifies the formation of AgNPs which correlated with the results obtained by (Prabu and Johnson, 2015; Santhoshkumar et al., 2021). The main reason behind the change in color is due to the resonance of surface plasmon by the electric fields of light (Liaqat et al., 2022). Further, the fabrication of AgNPs was analyzed by the peaks recorded by the UV–Vis spectrophotometer at 450 nm (Preda et al., 2020; Kalaiyarasan, 2022). AgNPs typically exhibit strong SPR bands in the visible spectrum between 400 and 500 nm. According to (El-Deeb et al., 2022) the size, type, temperature, shape, and environment of the surrounding particles, all have a significant impact on the surface plasmon resonance (SPR) absorbance.

In the present study, the absorption spectra indicate that AgNO_3_ concentrations; 1 mM, 2.5 mM, 5 mM, and 7.5 mM were inadequate for the synthesis of AgNPs as no peaks were observed whereas a sharp peak at 10 mM indicated as the optimal concentration for the phytofabrication of AgNPs. 10 mM is considered the ideal molarity for the present synthesis as the SPR lies well in the range of 450 nm which is well documented in the prior studies during the synthesis with *Hippophae rhamnoides* (Wei et al., 2020). The visual inspection from Figure 3A also strengthens the fact that there is no change in color from the concentration 1 mM to 7.5 mM to reddish brown which is the indicator of AgNPs synthesis and makes these particular concentrations inadequate for the present synthesis. Furthermore, the peaks of absorption spectra revealed that the optimal ratio of AgNO_3_ and plant extract was 9:1 for the effective synthesis of nanoparticles. The morphology and size of the nanoparticles are dependent upon the pH level of the solution. One of the key influences is the tendency of the reaction pH to change the electrical charges of biomolecules, which may affect their capacity for capping and stabilizing, in addition to the subsequent growth of the nanoparticles (Miranda et al., 2022). In the current study, there is no peak observed in acidic pH (2–6) which is also supported by visual examination as there is no distinct color change. According to Vanaja and Annadurai (2013), agglomeration occurs at low pH due to over-nucleation and the formation of larger nanoparticles. In our study, pH 8 is best suited for the biogenic synthesis of AgNPs and shows a sharp peak as compared to another alkaline pH like 9 and 10. pH has a pivotal role in the fabrication of nanoparticles and alkaline pH is known to facilitate the AgNPs formation (Handayani et al., 2020). The enhanced availability of functional groups at alkaline pH leads to faster nucleation and ultimately leads to the synthesis of nanoparticles (Kaur et al., 2023). The present results align with prior research indicating that phytofabrication of AgNPs is a pH-dependent reaction (Manosalva et al., 2019). Additionally, in the current study, at the alkaline pH 8, there is an instant synthesis of AgNPs at room temperature making the present synthesis energy efficient.

The FTIR spectrum shows that phytochemicals, particularly polyphenolic substances, and flavonoids in the extract, synthesize and stabilize AgNPs. Proteins are accountable for AgNP stabilization through capping which correlated with the results obtained by Jain and Mehata (2017) and Palithya et al. (2021). The FESEM results provided a clear insight into the size and shape of nanoparticles. The biogenic AgNPs were measured from 25–30 nm and were spherical in shape. The negative zeta potential value in the present investigation indicated that particles tend to repel each other and there will be no agglomeration among the nanoparticles. A negative zeta potential value of green synthesized AgNPs, suggests the presence of repulsive forces among the silver nanoparticles. This repulsion contributes to the enhanced stability of the formulation (Kotakadi et al., 2016; Elamawi et al., 2018). In XRD analysis, the crystalline size was evident and comparable to that of other plant-based AgNPs (Vasudeva et al., 2020).

The plant extract, AgNPs, and standard ascorbic acid all showed a dose-dependent inhibition of the DPPH radical in the scavenging assay. Pooled data from the DPPH assay revealed the lower IC_50_ value of AgNPs signifies them as a better antioxidant when compared to the plant extract. Silver ions and the present phytochemicals (flavonoids) could synergistically act as antioxidants by transferring a single electron and a hydrogen atom (Bedlovičová et al., 2020). The adsorption of phytochemicals on the surface of AgNPs might play a vital role in the better radical scavenging activity of AgNPs (Johnson et al., 2018). Lipid peroxidation is a free radical-initiated oxidative chain reaction in which one lipid molecule after another becomes oxidized to the maximum possible extent or to form lipid peroxide (Badmus et al., 2011). Research findings have demonstrated that lipid peroxidation exerts an influence on the fluidity of the cell membrane, hence compromising the structural integrity of the membrane and its components (Ammendolia et al., 2021; Sadžak et al., 2024). Malondialdehyde, the result of lipid peroxidation, prevents the synthesis of proteins and reacts with DNA bases (Park and Floyd, 1992; Seckin et al., 2022). Normally, this chain reaction is terminated when the substrate is depleted. Other condition includes the combination of two radicals to form a non-radical product or reaction with antioxidants, which provide easily donatable hydrogen for abstraction by peroxyl radicals (Upadhyay et al., 2014). In this study, AgNPs protect the egg yolk (lipid-rich media) from lipid peroxidation more effectively in contrast to the plant extract. The present study suggests that AgNPs could be used as a potential source of antioxidants. The antioxidant property of the biosynthesized AgNPs is due to the synergistic effect of silver ions and the presence of various phytocompounds in *Hippophae rhamnoides* (Tkacz et al., 2024), which in turn effectively inhibits lipid peroxidation by several chemical pathways such as radical addition, radical recombination, electron transport, and free radical scavenging during the lipid peroxidation propagation phase (Hodges et al., 1999; Abhinaya and Padmini, 2019). These results imply that AgNPs are important for preventing lipid peroxidation. Better inhibitory action of AgNPs by the virtue of quenching of the free radical, electron transfer, radical addition, or recombination effectively. The better antioxidant potential of AgNPs makes them better chain-breaking radical scavengers when compared to crude plant extract.

The results of the antimicrobial assay revealed that AgNPs fabricated from *Hippophae rhamnoides* berry extracts had a significant ability to inhibit both *S. aureus* and *E. coli*. The MIC data showed that the ideal AgNPs concentration is 1.0 mg/mL, with a maximal inhibition rate of 90.74% for *E. coli* and 87.37% for *S. aureus*. Concentrations less than 1.0 mg/mL could inhibit bacterial growth, although visible turbidity indicated insufficient inhibition. According to Gevorgyan et al. (2022) and Acharya et al. (2018), the interaction between silver nanoparticles (AgNPs) and the bacterial cell wall is reduced at low concentrations. The authors further claim that at higher concentrations, the antibacterial efficacy is further diminished due to lesser interactions with bacteria caused by the increased likelihood of NPs aggregation. The results are in concordance with our study where the concentration of less than as well as greater than 1.0 mg/mL showed less effect.

**Figure 13:**
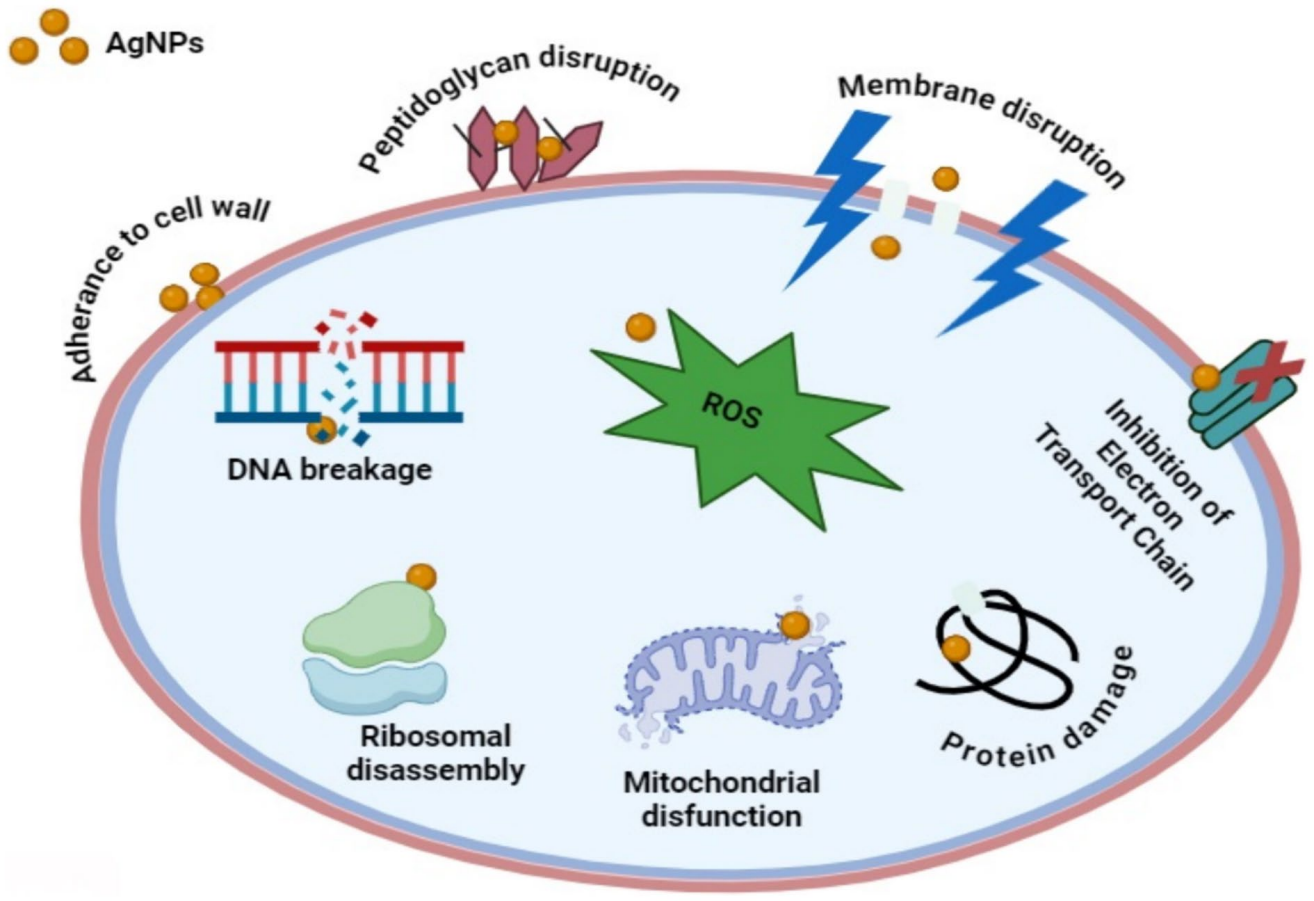
The antibacterial mechanisms of AgNPs involve multiple pathways. Firstly, AgNPs anchor to the bacterial cell wall, infiltrating it and causing membrane damage and leakage of cellular contents. This action can also involve AgNPs or Ag^+^ ions binding to membrane proteins involved in ATP generation, disrupting their function. Secondly, AgNPs penetrate microbial cells, where they release Ag^+^ ions interact with cellular structures and biomolecules such as proteins, enzymes, lipids, and DNA. This interaction leads to increased ROS production, causing an apoptosis-like response, lipid peroxidation, and DNA damage. Finally, AgNPs continuously release Ag^+^ ions both inside and outside the bacteria, with these ions interacting with proteins and enzymes, further contributing to the antimicrobial effect.

It is speculative that NPs could target microbial cell membranes, cause membrane potential damage, and ultimately kill bacterial cells (Slavin et al., 2017). In conjunction with the current work, silver nanoparticles synthesized from *Punica granatum* and *Ananas comosus* peel extract have been shown to have significant antibacterial activity (Jinu et al., 2017; Das et al., 2019). The present studies also revealed that the synthesized AgNPs possess better antibacterial activity against *E. coli* as compared to *S. aureus*. The results of Kim et al. (2007) and Feng et al. (2022) confirmed these findings where the antimicrobial efficacy of AgNPs was observed to be more pronounced against gram-negative compared to gram-positive bacteria. Due to the thick and hard peptidoglycan coating in the cellular wall, gram-positive bacteria are less likely to absorb AgNPs (Meikle et al., 2020; Yin et al., 2020). Numerous studies suggest that size has a greater influence on antimicrobial activity; the smaller the nanoparticle’s size, the greater its ability to penetrate microorganisms (Tang and Zheng, 2018; Roelofs et al., 2020; Marchidan et al., 2023). To elucidate the mechanism of antimicrobial activity, we have included a proposed hypothesis based on the current literature and our observations, which offers a plausible explanation for the antibacterial effects of AgNPs (Figure 13). AgNPs attach themselves to the membranes and cell walls of bacteria, altering their structure and permeability to cause cell destruction (Salleh et al., 2020). AgNPs produce reactive oxygen species (ROS), which lead to oxidative stress and subsequent cellular damage and death (Song et al., 2019). AgNPs also infiltrate cells and interfere with DNA replication and other protein processes, which worsens and ultimately kills bacterial cells (Yamanaka et al., 2005).

## 5 Conclusion

Due to eco-rich and leading-edge applications, green nanotechnology-based nanostructures have become increasingly attractive in the biomedical and pharmaceutical industries. The mining of natural resources has created numerous opportunities for the environmentally sustainable synthesis of nanoparticles. Our study demonstrates the utilization of ethnomedical sea buckthorn berries for one-pot synthesis of AgNPs. The phytochemicals present in berries result in the rapid reduction of silver salts to silver nanoparticles. The characterization of biosynthesized AgNPs showed a range of particle sizes from 25–30 nm and the particles were isotropic and crystalline in nature. The biosynthesized-mediated AgNPs showed remarkable free radical scavenging potential. The process of lipid peroxidation was also retarded by the AgNPs. The AgNPs produced through biogenic synthesis demonstrated antibacterial activity against both gram-positive and gram-negative bacteria.

## Data availability statement

The original contributions presented in the study are included in the article/supplementary material, further inquiries can be directed to the corresponding authors.

## Author contributions

NR: Writing – original draft. AB: Writing – review & editing. BK: Resources, Writing – review & editing. SS: Resources, Visualization, Writing – review & editing. NA-r: Writing – review & editing. NJ: Formal analysis, Writing – review & editing. FB: Investigation, Methodology, Writing – review & editing. EV: Funding acquisition, Writing – review & editing. MS: Project administration, Writing – review & editing.
